# Evidence for the Concreteness of Abstract Language: A Meta-Analysis of Neuroimaging Studies

**DOI:** 10.3390/brainsci12010032

**Published:** 2021-12-28

**Authors:** Nicola Del Maschio, Davide Fedeli, Gioacchino Garofalo, Giovanni Buccino

**Affiliations:** 1 Faculty of Psychology, Università Vita-Salute San Raffaele, 20132 Milano, Italy; delmaschio.nicola@hsr.it (N.D.M.); d.fedeli@studenti.unisr.it (D.F.); 2 Divisione di Neuroscienze, Università Vita-Salute San Raffaele, 20132 Milano, Italy; g.garofalo@docenti.unisr.it; 3 IRCCS San Raffaele, 20132 Milano, Italy

**Keywords:** abstract concepts, embodiment, meta-analysis, Broca’s region, neuroimaging

## Abstract

The neural mechanisms subserving the processing of abstract concepts remain largely debated. Even within the embodiment theoretical framework, most authors suggest that abstract concepts are coded in a linguistic propositional format, although they do not completely deny the role of sensorimotor and emotional experiences in coding it. To our knowledge, only one recent proposal puts forward that the processing of concrete and abstract concepts relies on the same mechanisms, with the only difference being in the complexity of the underlying experiences. In this paper, we performed a meta-analysis using the Activation Likelihood Estimates (ALE) method on 33 functional neuroimaging studies that considered activations related to abstract and concrete concepts. The results suggest that (1) concrete and abstract concepts share the recruitment of the temporo-fronto-parietal circuits normally involved in the interactions with the physical world, (2) processing concrete concepts recruits fronto-parietal areas better than abstract concepts, and (3) abstract concepts recruit Broca’s region more strongly than concrete ones. Based on anatomical and physiological evidence, Broca’s region is not only a linguistic region mainly devoted to speech production, but it is endowed with complex motor representations of different biological effectors. Hence, we propose that the stronger recruitment of this region for abstract concepts is expression of the complex sensorimotor experiences underlying it, rather than evidence of a purely linguistic format of its processing.

## 1. Introduction

The embodied approach to language claims that the same neural structures involved in making sensory, motor, and even emotional experiences are also involved in understanding the linguistic material related to those experiences [1,2,3,4,5,6,7,8,9,10,11,12,13]. At least for language-expressing concrete content, such as nouns of graspable objects or action verbs, several experimental findings have supported this theoretical framework [14,15,16,17,18,19,20,21,22,23,24,25,26,27,28,29,30,31,32], for a review see [6,33] However, the embodiment provides less straightforward results in the domain of abstract concepts. Indeed, by definition, abstract concepts are far from actual experiences, so they can appear to be hardly rooted in the neural substrates subserving those experiences.

Abstract concepts have been defined as mental representations referring to entities that are neither purely physical nor spatially constrained [34]. When compared with concrete concepts like “apple” or “table”, abstract concepts like “truth” or “freedom” are generally more variable in their content across individuals and more difficult to associate with a single image. Indeed, abstract and concrete concepts are expressed by words (or combinations of words) that are used to refer to complex mental states (e.g., thought and happiness), conditions (uncertainty), situations (encounters), and relationships (employment), or more simply to denote material objects [33,35,36,37] (for a philosophical discussion on this point, see below the view of Locke). For consistency, throughout the paper, we will use the notion of abstract and concrete concepts, although in the different experiments authors used verbal stimuli defining those concepts.

According to Paivio’s “dual-coding” theory [38,39,40], the fundamental difference between abstract and concrete concepts is that the former would rely solely on verbal representations, while the latter would evoke both verbal and visuo-perceptual ones. In Paivio’s hypothesis, cognitive processes involve the activity of a verbal system (located in the language-dominant hemisphere) and an “imagery” system dedicated to real objects and events (spread across both hemispheres). These two systems are based upon distinct and modality-specific representation units: the “logogens” for the verbal system and the “imagens” for the imagery system. Given the difficulty in imaging abstract concepts, the dual-coding theory claims that abstract concepts are represented only through logogens, while concrete concepts would activate both logogens and imagens, thus having a dual codification. The dual codification of concrete concepts, which involves the additional contribution of the imagery system, would account for the so-called “concreteness effect”, according to which concrete words have the advantage over abstract ones in terms of both recalling and recognition [41,42,43].

Even within scholars that contributed to developing the embodied approach to language, abstract concepts remain a topic of great debate. In this respect, Dove [44] defined the limits that abstract concepts pose for embodiment: (1) the problem of generalization, which is the capability of building super-ordinate concepts encompassing several subordinate ones; (2) the problem of flexibility, which is the fact that a number of factors (e.g., physical environments, situations, body states, and current tasks) may affect the way concepts are realized; and (3) the problem of disembodiment; that is, there may be contexts in which the embodiment of some notions is not ascertained immediately, like high-level mathematical notions [45,46].

Some authors have also stressed the emotional valence of abstract concepts [1,34,47,48,49]. Vigliocco and colleagues [48,49], for instance, have proposed that the meaning of abstract concepts can be mainly grounded in emotional experiences rather than in sensorimotor ones. The perception of internal states of the organism (e.g., an acceleration in the heart’s pace or a sudden sweat) in response to certain environmental stimuli (e.g., seeing a predatory animal or receiving a harsh scolding from one’s boss) is at the basis of emotional experiences (e.g., fear or shame). Accordingly, the meaning of abstract concepts would be rooted in neural substrates involved in processing these internal and emotional states. Evidence in this direction comes from the results of Vigliocco et al. [47], who showed a correlation between the degree of activation of the anterior cingulate, an area that has been related to emotion processing [50,51], and the level of emotional charge of the verbal stimuli (i.e., the induced pleasant or unpleasant feelings).

Another proposal, known as the “Words as Tools” (WAT) approach [35,52,53,54], shares with the general embodied approach the idea that all words are grounded in the neural substrates subserving motor, sensorial, and emotional experiences, but it also puts forward that abstract concepts partially differ from concrete ones. The WAT approach stresses that concrete and abstract concepts are learned in different contexts and at different ages. While the meaning of a concrete word usually derives from the direct interaction with the word’s referent (an object or an event), the situation would be different for abstract words like “God” or “virtue”. According to WAT, the meaning of the word “God” is not primarily grounded in the direct experience that somebody can make of God but rather following the conventions established by the social context in which individuals live, act, and speak and eventually on the verbal interactions that take place in such contexts. Collectively shared rules are the origin of the meaning of such abstract words. Hence, these rules lead the individuals to select a set of bodily states (as well as of internal and external experiences) that come to define the meaning of a certain abstract word. The meaning of abstract words is rooted in the social use of these words, and therefore, each individual learns the meaning from the social context to which he or she is exposed. In this sense, abstract words are social tools. Consequently, the WAT approach suggests a clear distinction between concrete and abstract words, in which the first mainly rely on perception and action, whereas the second are mainly based on social sharing and primarily coded by a dedicated language system [54,55].

As a whole, reviewing the theoretical frameworks proposed to explain how the brain codes abstract concepts unveils that, even among the supporters of embodiment, there is not a unique view for concrete concepts. Moreover, in no case are specific neural substrates and mechanisms considered the only elements necessary and sufficient to process abstract concepts. Aside from and beyond modal aspects, additional amodal aspects and mechanisms are evoked to fully grasp them. As underlined in some recent reviews on this topic [44,56,57,58,59], hybrid models that take into account modal and amodal aspects are generally considered better means to explain the processing of abstract concepts.

To the best of our knowledge, only one recent proposal [60] has suggested a “strong” embodied version to process abstract concepts, ruling out the role of hybrid models. This proposal assumes that abstract concepts differ from concrete ones not because they are disentangled from experiences or acquired in social interactions, but rather because they are grounded in more complex sensory, motor, and emotional experiences compared with concrete concepts. The authors define this complexity in three main points: (1) abstract concepts are represented by different biological effectors (abstract meaning as effector-unspecific); (2) they recruit different systems, including sensory, motor, and emotional ones (abstract meaning as multi-systemic); and (3) they change over time and across cultures (abstract meaning as dynamic), and hence the neural substrate coding for social contexts and levels of self-relatedness should be more strongly involved in processing these concepts compared with concrete ones.

It is worth noting that, in the philosophical domain, this approach is not completely new and is reminiscent of Locke’s view [61] on ideas and thoughts. The philosopher states that the only basis of ideas and thoughts is experience, including external sensations (i.e., the affection that external reality exerts on our senses) and internal reflections (i.e., the mind’s consideration of its own operations). In Locke’s view, ideas can be divided into simple ideas and complex ones. Simple ideas correspond to elementary aspects of external reality as grasped by our senses, such as the “coldness of ice” or the “sweetness of sugar”. Complex ideas such as “beauty”, “gratitude” and so on include many different simple ideas already acquired from experience. Therefore, in Locke’s view, complex ideas have the same origin as the simple ones (i.e., experience). Complex ideas are such because of the complexity of the experiences they refer to. It is because of this complexity that these ideas are apparently further from experience than concrete ones.

According to Locke, words are signs of ideas. In this respect, the author distinguishes proper names (e.g., “Mount Everest”) from general words (e.g., “mountain”). This latter category includes words expressing simple and complex ideas (as defined above). Thus, the words we define as concrete and abstract are included in the category of general words by Locke. Accordingly, the meanings of general words, both concrete and abstract, are always grounded in the experience they point at. The only difference is that the experience is simpler in the case of concrete words and more complex for abstract words expressing complex ideas.

The aim of the present meta-analysis is to assess whether (and to what extent) the processing of concrete and abstract concepts is subserved by the same neural structures. The meta-analytic results will be discussed in light of the hypothesis that abstract concepts are more complex than concrete ones, rather than disentangled from actual experiences.

## 2. Materials and Methods

### 2.1. Data Collection and Preparation

**Figure 1:**
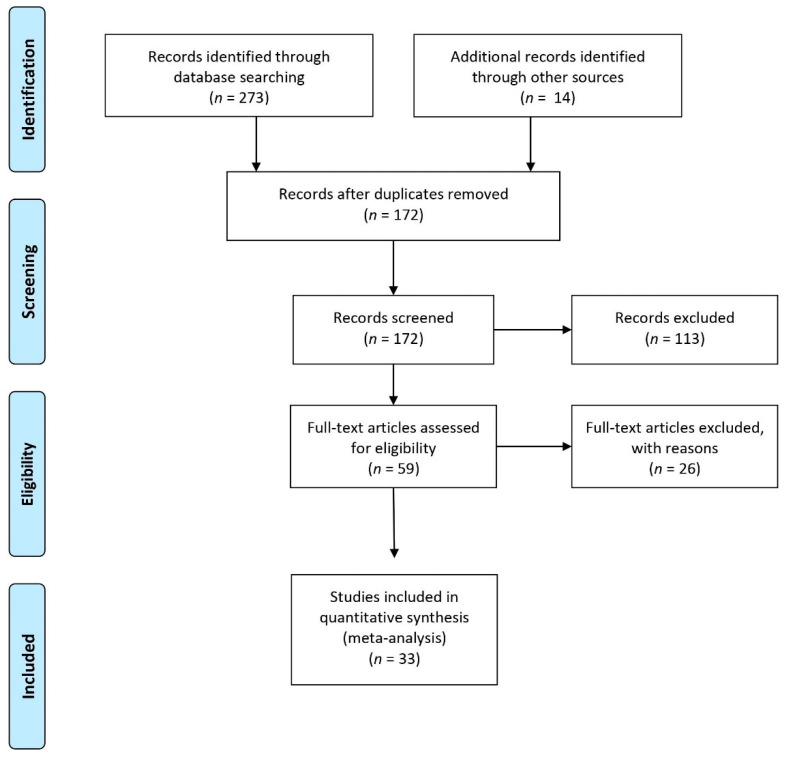
Preferred Reporting Items for Systematic Reviews and Meta-Analyses (PRISMA) flow diagram of the literature search (http://www.prismastatement.org/, accessed on 25 April 2020).

The current meta-analysis was based on the Preferred Reporting Items for Systematic Reviews and Meta-Analyses (PRISMA) Statement guidelines (http://www.prisma-statement.org/, accessed on 25 April 2020). PRISMA guidelines suggest following a 27-item checklist and reporting a flow diagram of the literature search and paper inclusion (see Figure 1).

We performed a set of coordinate-based meta-analyses of functional neuroimaging studies investigating the processing of concrete and abstract concepts in healthy individuals. Articles were selected through an online literature search in the Scopus, Pubmed, and Web of Science databases using the following input search keywords: “((Abstract words AND abstract concepts) and (fMRI OR PET OR neuroimaging OR brain imaging))”. Only studies written in English and published from January 1990 to April 2020 were included. This preliminary search returned a total of 272 results, and 14 additional studies were identified through other sources i.e. [62], yielding a total of 286 results. Duplicates were removed from this initial set leading to a total of 172 results. On the basis of the title and abstract, a first screening was independently conducted by two authors (N.D.M. and F.D.) based on the following inclusion criteria: (1) peer-reviewed published journal articles; (2) functional magnetic resonance imaging (fMRI) or positron emission tomography (PET) studies; and (3) healthy monolingual adults (18–35 years). Unagreed cases were discussed and eventually resolved by the two authors who performed the screening, leading to a total of 59 eligible articles. These articles were then read in full to verify whether they could be included in the final sample. During this second screening stage, a further inclusion criterion was added: (4) reported activation for concrete words or sentences > abstract words or sentences and abstract words or sentences > concrete words or sentences. At this screening stage, further exclusion criteria were applied to identify only those studies reporting the spatial coordinates of the activation patterns for concrete and abstract concept processing. Excluded were (1) review or meta-analysis studies, (2) studies with an absence of coordinates reported from whole-brain activation in Talairach [63] or the Montreal Neurological Institute MNI, [64] stereotaxic space (i.e., regions-of-interest-based studies or small-volume corrections applied to the analysis), (3) studies of brain connectivity (e.g., resting-state fMRI and multi-voxel pattern analysis), (4) results from tasks using experimental stimuli other than linguistic ones (e.g., images or pictograms). The final sample included 33 articles (see Figure 1). We assumed that these studies were approved by their respective ethics committees prior to data collection.

### 2.2. Data Classification

The reported coordinates were extracted and divided into 4 main sets based on our contrasts of interest: (1) main effect of abstractness, (2) main effect of concreteness, (3) abstract > concrete, and (4) concrete > abstract (for a detailed list of the experimental tasks included in each contrast, see Table 1). By considering these contrasts, we collapsed the studies using either single words or sentences for both the concrete and abstract concepts. All group contrasts included a sufficient number of peaks in accordance with the power guidelines [65].

### 2.3. Data Analysis

We performed analyses for each subset of coordinates through the GingerALE software (Brainmap GingerALE version 2.3.6; Research Imaging Institute, San Antonio, TX, USA) using the Activation Likelihood Estimation (ALE) method and non-additive correction to minimize the within-experiment effects as described in Turkeltaub et al. [97,98]. GingerALE takes the peaks of the activation coordinates from neuroimaging studies, applies an inclusive brain mask and a subject-size-based full width at half maximum (FWHM), estimates the probability of the coordinates’ spatial distribution, and then computes the convergence of these probabilities. The coordinates reported in Talairach space were first converted to MNI space with the GingerALE conversion tool. The number of participants was then specified for each selected contrast, with this parameter being required for estimating the FWHM of the Gaussian function used during ALE maps estimation [99]. For each subset, the analyses were performed with thresholding ALE maps at uncorrected *p* < 0.001 and by setting a minimum cluster size of 150 mm^3^ (the same cluster extent threshold has been adopted by several other ALE meta-analyses [100,101,102]. Additionally, family-wise error (FWE) cluster level corrected *p* < 0.05 and *p* < 0.001 peak level uncorrected analyses were performed, and clusters surviving this threshold are highlighted in the result tables. A conjunction analysis between the sets of (1) the main effect of abstractness and (2) the main effect of concreteness was performed in order to show areas of spatial overlay. The clusters in each set were thresholded at *p* < 0.05 uncorrected and entered into the conjunction analysis, which was thresholded with a *p* < 0.05 false discovery rate (FDR) correction.

## 3. Results

### 3.1. Abstract ∩ Concrete

The conjunction analysis between concreteness and abstractness revealed extensive clusters in the left temporal lobe, including the middle and inferior temporal gyri, and in the left motor cortex. Significant activations were also found in the right parietal cortex, left inferior frontal gyrus, and prefrontal regions (see Table 2).

### 3.2. Concrete > Abstract

The concrete > abstract contrast revealed left parietal (inferior parietal lobule, angular, and supramarginal gyri), temporal (fusiform and middle temporal gyri), and posterior (precuneus) activations. Smaller clusters included the left inferior frontal gyrus and the middle cingulate cortex (see Table 3).

### 3.3. Abstract > Concrete

**Figure 2:**
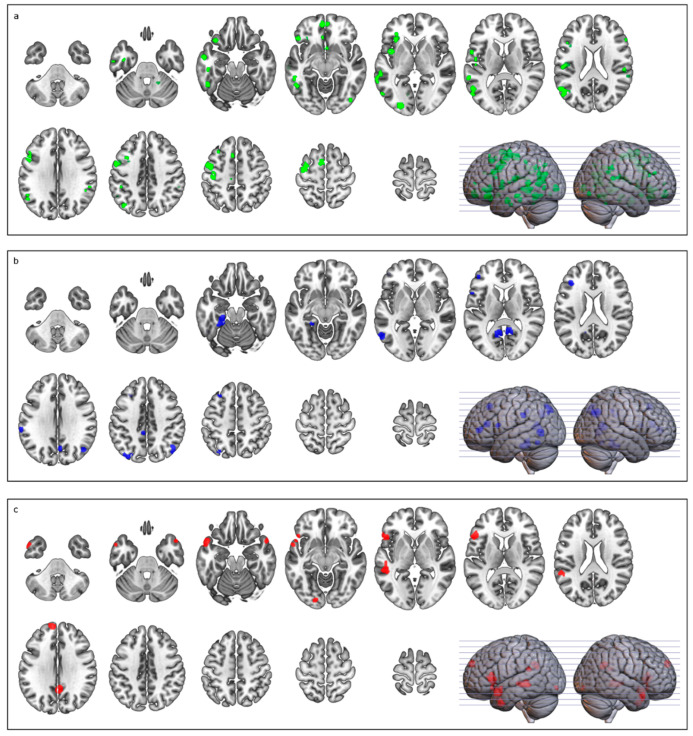
Brain activation related to (**a**) conjunction analysis (green); (**b**) concrete > abstract (blue); and (**c**) abstract > concrete (red). Images are in neurological display convention.

The abstract > concrete contrast revealed two major clusters situated on the left inferior frontal gyrus (pars triangularis and orbitalis) and middle temporal gyrus. Smaller clusters included the medial frontal cortex and the bilateral temporal poles (see Table 4 and Figure 2).

## 4. Discussion

The present meta-analysis included 33 articles published over the last 30 years. We will discuss the results in the following order: (1) common brain activation related to concrete and abstract concepts, (2) brain activation when contrasting concrete > abstract, and finally (3) brain activation when contrasting abstract > concrete.

Common activation for concrete and abstract concepts included areas within the frontal lobe in the motor and premotor cortex (i.e., left precentral and postcentral gyri, supplementary motor area, and inferior frontal gyrus), areas in the parietal lobe (i.e., the right supramarginal gyrus), and areas in the temporal lobe (i.e., the left middle and inferior temporal gyri). These results suggest that both concrete and abstract concepts recruit temporo-fronto-parietal circuits normally involved when individuals interact with objects and, more generally, with the environment [103,104,105,106,107,108]. In fact, these findings are not surprising when considering concrete words. Several studies have shown an activation of the fronto-parietal areas during the processing of verbs, e.g., [16,23,68,77,78,79,80,81,109,110,111,112,113,114,115] and nouns [17,27,66,68,116] expressing a specific motor content. However, at first glance, the results are unexpected when considering abstract concepts. Even within the embodiment literature [33,53,56,58,117], abstract concepts are often considered disentangled from sensorimotor experiences and mainly processed in the so-called “language system”. It is not always clear, however, whether in the current literature the notion of a language system refers to the inferior frontal gyrus (IFG) including Broca’s region [44,52] or to specific high-order regions, namely the “semantic hubs” [33,58,118,119]. Whatever the brain structures involved, this conjunction analysis challenges the proposal that abstract concepts are exclusively coded in a propositional format [44,52], in semantic hubs [33,58,118,119], or, at most, in areas related to emotions [1,47,49]. Rather, it supports the notion that, like concrete concepts, abstract concepts are grounded in neural structures where sensory, motor, and emotional experiences are coded.

It turns out that abstract concepts are not such because their meaning is “far from experience”, but rather that the concrete experiences expressed by abstract concepts are similar to those underlying concrete language, although they possibly have a higher complexity that is grounded in different neural systems (multi-systemic), involves different effectors (e.g., the hand, mouth, and possibly foot), and evolves dynamically across the lifespan [60]. In keeping with this interpretation of the present meta-analytic results, at the behavioral level, there is evidence that the concreteness effect [120]—that is, a facilitation of semantic processing of concrete words compared with abstract words—decreases when abstract words are contextualized [121,122]. This in turn suggests that abstract concepts are also rooted in concrete experiences and grounded in brain areas underlying those experiences.

When comparing concrete > abstract concepts, the present meta-analysis showed activation in the temporo-parietal areas (angular gyrus; supramarginal gyrus), posterior cingulate and parahippocampal gyrus, and precuneus. Activations in these regions have already been found in previous meta-analyses on the matter [62,117]. In addition, the present study showed an activation of the premotor areas (including the IFG). The activations of posterior areas have been explained as being due to motor and visual imagery [62], which indeed could be more salient in concrete than abstract concepts. Aside from these posterior activations, sectors of the motor and premotor cortex (inferior parietal lobule (IFG)) were also active. Parietal areas are strictly connected with frontal ones in sensorimotor circuits, which are involved in the sensorimotor transformation and manipulation of objects as well as in processing language with motor content [20,105,106,107,108,123,124,125,126]. On these grounds, we put forward that the presence of activation in the parietal and premotor sectors could reflect a stronger and more specific recruitment of these sensorimotor circuits during the processing of concrete concepts. In this respect, it is worth remarking that the majority of studies included in this meta-analysis used, as experimental stimuli, nouns referring to graspable objects and tools and, in a few cases, sentences expressing goal-directed actions.

As for mesial activation (i.e., parahippocampal gyrus and posterior cingulate), these areas were also found by Binder et al. [117]. Classically, these areas are considered to be involved in semantic and episodic memory, e.g., [127,128]. Following the interpretation of Binder et al. [85], these areas may act as an interface between the semantic retrieval and episodic encoding systems. Specifically, their role could be to form a record of past experience to be used for guiding future behavior. We extend this interpretation by proposing that the role of such areas is to select and recall the most relevant experiences to be motorically re-enacted by the fronto-parietal circuits in order to provide meaning and contextualize lexical items.

Finally, the angular gyrus (AG) has been involved in several functions, including semantic processing, word reading and comprehension, memory retrieval, self-processing, attention and spatial cognition, and social cognition (for a review see [129]). The activation of this area in the concrete > abstract contrast is quite surprising, since this area also has a role in processing abstract concepts, and it is usually indicated as one of the semantic hubs [58]. What is relevant with respect to our hypothesis concerning the distinction between abstract and concrete concepts is the evidence that the AG is an area also involved in the execution, observation, and imagination of non-object directed actions [130,131,132]. It seems that the AG codes the motor aspects of an action even in the absence of a specific object upon which the action is performed. Although there is no doubt that this area has a putative role in different cognitive processes, we propose that the AG may code for motor aspects that are common to different kinds of actions. The present results further suggest this interpretation, supporting the view that this area could provide a general description of an action even when expressed verbally. In other words, this area could have the role of synthetizing concrete experiences. In this respect, it seems to code abstraction rather than abstract concepts.

The abstract > concrete comparison showed activation of the IFG and the middle temporal gyrus. These activations largely overlap with those found by previous meta-analyses [57,62,117,133]. The activation of the IFG has been related to verbally mediated semantic knowledge processing [134,135]. Binder et al. [66] suggested that the stronger activation of the left IFG during a lexical decision task reflects the additional semantic processing for abstract vs. concrete words, as the former would be held in working memory in phonological form to a greater degree than concrete words. As a whole, this prevalent activation of the IFG during abstract vs. concrete concept processing seems to fit with the general notion of a major involvement of the language system in the processing of abstract concepts [44,52,58,136]. It has been proposed that the involvement of this region in processing abstract concepts could be largely justified by the acquisition modality of abstract concepts. Concept acquisition would differ for concrete and abstract concepts because only concrete concepts would be learned via sensory experience with the physical world, while abstract concepts would be acquired through their use in sentences and their relationships to other concepts. Therefore, the representation format would vary, with concrete concepts (i.e., the meanings of concrete words) being represented in visual, auditory, tactile, or gustatory formats and abstract concepts (i.e., the meanings of abstract words) represented in a propositional format. The specification of meaning would vary because concrete concepts correspond directly with entities in the physical world and have a fixed core meaning, while the meanings of abstract concepts would be largely specified by the sentence context (e.g., “the phase of the moon” or “the phase of development”) [52,121,122]. Since linguistic experience is crucial for the acquisition and representation of abstract concepts, following this view, the major involvement of the IFG in abstract concept processing may be justified by the fact that this sector of the brain largely coincides with Broca’s area [137], a region classically devoted to speech production and endowed with a mouth motor representation that possibly also includes the control of all communicative acts necessary for building up the linguistic experience at the basis of abstract concept acquisition and processing.

In summary, while the meanings of concrete concepts are supposed to be defined by perceptual features and their relation to the physical world, the meanings of abstract concepts are thought to be verbally mediated and emerge from use in sentence contexts [94].

In contrast with the classical view of Broca’s region as a pure linguistic area, several anatomical and neurophysiological findings have clearly demonstrated that the IFG, where Broca’s region lies, is not only endowed with mouth motor representation but also includes hand–arm representation. Potentially all biological effectors are represented in the IFG, both in monkeys as well as in humans [106,138,139,140,141,142]. In the monkey, the IFG contains neurons (known as mirror neurons) that discharge not only during the execution of actions but also during the mere observation of the same or a similar action or during listening to a sound usually associated with those actions [140,143]. The human homologue of the IFG, Broca’s region, is involved in goal-directed actions [138], action observation e.g., [116,144,145,146,147] (for a review see [117]), motor imagery, and action imitation (for a review see [148]). Furthermore, in human individuals, the IFG is also involved in coding hand actions even when disentangled from the use of objects, like in mimicked actions, meaningless actions, and emblems [130,131,132]. Overall, it appears that in Broca’s region there is a representation of actions carried out with different biological effectors (the hand, mouth, and possibly foot). What is relevant in the present context is that apparently these motor representations are active not only when actions are actually performed but also when they are recognized or imagined. In a sense, we could speak of a conceptual representation of concrete actions.

It has been put forward that in the phylogenesis, as well as in cultural evolution [45,141,149], the involvement of Broca’s region in verbally mediated semantic processing could derive from this conceptual use and representation of actions.

On the basis of this empirical evidence, we put forward that the prevalent activation of the IFG during the processing of abstract vs. concrete concepts may indicate that the recruitment of this area is related to a re-enactment of actions unchained from the use of specific objects and from specific contexts, rather than due to the understanding of abstract concepts in a purely propositional linguistic format. Put differently, because abstract concepts and their corresponding verbal labels express actions or entities that are dynamic in time and space and may be executed by different effectors and coded in different systems [60], their content, more strongly than for concrete concepts and words, is coded “motorically” in a brain region where actions are represented in a conceptual manner.

Of note, the findings reported here and the interpretation proposed should be considered in light of a number of limitations. In particular, the meta-analytic power of the present study is intrinsically limited by the amount of available data, especially for the concrete > abstract analysis. Therefore, if sufficiently powered to detect large summary effect sizes, the present study might have failed to detect smaller effects.

## 5. Conclusions

Overall, the present meta-analysis suggests that abstract concepts and concrete concepts share common neural substrates, including not only areas classically considered semantic hubs but also parieto-frontal structures involved in coding actions. These findings may be interpreted as indirect evidence against the notion of abstract concepts disentangled from sensorimotor experiences. As proposed in a recent review [60], the sensorimotor experiences underlying abstract concepts are even more complex than those expressed by concrete ones because they can involve different effectors and systems. The stronger activation of Broca’s region in processing abstract vs. concrete concepts may further support this view. In fact, this region (classically considered part of the language network) is endowed with motor representations related to different effectors and disentangled from object use. In other words, Broca’s region may also code for a complex, abstract, and synthetic representation of actions. On these grounds, rather than coding abstract concepts in a purely propositional format [44,52,56,62,94,117,119], we put forward that this region codes for the complexity of sensorimotor experiences (unchained from the use of specific objects and contexts) in which abstract concepts are grounded. Different from abstract concepts, language expressing concrete contents activates more strongly parieto-frontal circuits known to be involved in coding the pragmatic and perceptual features of an object and the motor aspects to interact with them [105,108,150,151,152].

## Figures and Tables

**Table 1:** Descriptive information of the 33 experiments included in the meta-analysis. Reported coordinates were extracted and divided into 4 main sets based on the contrasts of interest. Language: languages used for the creation of experimental stimuli in each study. Stimuli: N = noun; V = verb: ADJ = adjective.

**Main Effect Concrete (Conjunction)**									
**Study (*n* = 13)**	**Subjects**	**Task**	**Language**	**Method**	**Design**	**Stimuli**	**Modality**	**Contrast**	**195 foci**
[66] Binder et al., 2005	24	lexical decision	English	fMRI	event-related	word (N)	visual	concrete > pseudoword	39
[67] Bonner et al., 2013	20	lexical decision	English	fMRI	block	word (N)	visual	concrete > pseudoword	19
[68] Della Rosa et al., 2018	27	lexical decision	Italian	fMRI	event-related	word (N)	visual	high imageability, high context availability	9
[69] Dreyer et al., 2018	28	passive reading	German	fMRI	event-related	word (N)	visual	concrete > baseline (fixation)	11
[70] González et al., 2006	23	passive reading	Spanish	fMRI	block	word (N, ADJ)	visual	concrete > baseline (implicit)	8
[71] Grossman et al., 2002	16	semantic judgment (pleasant or not)	English	fMRI	block	word (N)	visual	concrete > pseudoword	3
[72] Hayashi et al., 2014	16	passive reading	Japanese	fMRI	block	word (N)	visual	concrete > baseline (fixation)	13
[73] Kiehl et al., 1999	6	lexical decision	English	fMRI	block	word (N)	visual	concrete > baseline (fixation)	19
[74] Raposo et al., 2006	15	lexical decision	English	fMRI	event-related	word (N)	visual	concrete > baseline (implicit)	33
[75] Roxbury et al., 2014	17	lexical decision	English	fMRI	event-related	word (N)	auditory	concrete > pseudoword	4
[76] Skipper et al., 2014	19	semantic judgment (semantic questions)	English	fMRI	block	word (N)	visual	concrete > pseudoword	14
[77] Tettamanti et al., 2008	18	passive listening	Italian	fMRI	event-related	sentence	auditory	concrete > baseline (implicit)	16
[47] Vigliocco et al., 2014	20	lexical decision	English	fMRI	block	word (N)	visual	concrete > pseudoword	7
**Main Effect Abstract (Conjunction)**									
**Study (*n* = 15)**	**Subjects**	**Task**	**Language**	**Method**	**Design**	**Stimuli**	**Modality**	**Contrast**	**225 foci**
[66] Binder et al., 2005	24	lexical decision	English	fMRI	event-related	word (N)	visual	abstract > pseudoword	23
[67] Bonner et al., 2013	20	lexical decision	English	fMRI	block	word (N)	visual	abstract > pseudoword	6
[68] Della Rosa et al., 2018	27	lexical decision	Italian	fMRI	event-related	word (N)	visual	low imageability, low context availability	32
[69] Dreyer et al., 2018	28	passive reading	German	fMRI	event-related	word (N)	visual	abstract > baseline (fixation)	8
[71] Grossman et al., 2002	16	semantic judgment (pleasant or not)	English	fMRI	block	word (N)	visual	abstract > pseudoword	3
[78] Harpaintner et al., 2020	24	lexical decision	German	fMRI	event-related	word (N)	visual	abstract > baseline (implicit)	30
[72] Hayashi et al., 2014	16	passive reading	Japanese	fMRI	block	word (N)	visual	abstract > baseline (fixation)	9
[73] Kiehl et al., 1999	6	lexical decision	English	fMRI	block	word (N)	visual	abstract > baseline (fixation)	19
[79] Ortigue et al., 2007	36	lexical decision	English	fMRI	block	word (N)	visual	abstract > baseline (implicit)	26
[74] Raposo et al., 2006	15	lexical decision	English	fMRI	event-related	word (N)	visual	abstract > baseline (implicit)	3
[75] Roxbury et al., 2014	17	lexical decision	English	fMRI	event-related	word (N)	auditory	abstract > pseudoword	3
[80] Saxbe et al., 2013	28	emotional rating of videos	English	fMRI	block	sentence	visual or auditory	abstract > baseline (implicit)	32
[76] Skipper et al., 2014	19	semantic judgment (semantic questions)	English	fMRI	block	word (N)	visual	abstract > pseudoword	19
[77] Tettamanti et al., 2008	18	passive listening	Italian	fMRI	event-related	sentence	auditory	abstract > baseline (implicit)	5
[47] Vigliocco et al., 2014	20	lexical decision	English	fMRI	block	word (N)	visual	abstract > pseudoword	7
**Concrete > Abstract**									
**Study (*n* = 15)**	**Subjects**	**Task**	**Language**	**Method**	**Design**	**Stimuli**	**Modality**	**Contrast**	**132 foci**
[66] Binder et al., 2005	24	lexical decision	English	fMRI	event-related	word (N)	visual	concrete > abstract	15
[81] Fiebach et al., 2004	12	lexical decision	German	fMRI	event-related	word (N)	visual	concrete > abstract	1
[82] Fliessbach et al., 2006	21	word recognition	German	fMRI	event-related	word (N)	visual	concrete > abstract	3
[83] Giesbrecht et al., 2004	10	semantic judgment (word-relatedness)	English	fMRI	event-related	word (N)	visual	high imageability > low imageability	4
[84] Harris et al., 2006	20	semantic judgment (pleasant or not)	English	fMRI	block	word (N)	visual	concrete > abstract	8
[72] Hayashi et al., 2014	16	passive reading	Japanese	fMRI	block	word (N)	visual	concrete > abstract	7
[85] Hoffman et al., 2015	20	semantic judgment (synonym judgment)	English	fMRI	block	word (N)	visual	concrete > abstract	13
[86] Jessen et al., 2000	14	memory encoding	German	fMRI	block	word (N)	visual	concrete > abstract	13
[75] Roxbury et al., 2014	17	lexical decision	English	fMRI	event-related	word (N)	auditory	concrete > abstract	5
[87] Sabsevitz et al., 2005	28	semantic judgment (semantic similarity)	English	fMRI	event-related	word (N)	visual	concrete > abstract	26
[88] Straube et al., 2013	20	semantic judgment (content judgment)	German, Russian (control)	fMRI	event-related	sentence	visual or auditory	concrete > abstract	1
[23] Tettamanti et al., 2005	17	passive listening	Italian	fMRI	block	sentence	auditory	concrete > abstract	19
[89] Van Dam et al., 2016	14	passive reading	English	fMRI	event-related	sentence	visual	concrete > abstract	4
[90] Wallentin et al., 2005	18	semantic judgment (comprehension)	Danish	fMRI	block	sentence	visual or auditory	concrete > abstract	10
[91] Wilson-Mendenhall et al., 2013	13	semantic judgment (concept-scene match)	English	fMRI	block	word (N, V)	visual	concrete > abstract	3
**Abstract > Concrete**									
**Study (*n* = 22)**	**Subjects**	**Task**	**Language**	**Method**	**Design**	**Stimuli**	**Modality**	**Contrast**	**146 foci**
[66] Binder et al., 2005	24	lexical decision	English	fMRI	event-related	word (N)	visual	abstract > concrete	9
[92] Binney et al., 2016	19	semantic judgment (forced choice)	English	fMRI	block	word (N, ADJ)	visual	abstract > concrete	12
[68] Della Rosa et al., 2018	27	lexical decision	Italian	fMRI	event-related	word (N)	visual	abstract > concrete	1
[81] Fiebach et al., 2004	12	lexical decision	German	fMRI	event-related	word (N)	visual	abstract > concrete	1
[82] Fliessbach et al., 2006	21	word recognition	German	fMRI	event-related	word (N)	visual	abstract > concrete	2
[71] Grossman et al., 2002	16	semantic judgment (pleasant or not)	English	fMRI	block	word (N)	visual	abstract > concrete	5
[84] Harris et al., 2006	20	semantic judgment (pleasant or not)	English	fMRI	block	word (N)	visual	abstract > concrete	2
[72] Hayashi et al., 2014	16	passive reading	Japanese	fMRI	block	word (N)	visual	abstract > concrete	2
[85] Hoffman et al., 2015	20	semantic judgment (synonym judgment)	English	fMRI	block	word (N)	visual	abstract > concrete	21
[86] Jessen et al., 2000	14	memory encoding	German	fMRI	block	word (N)	visual	abstract > concrete	2
[73] Kiehl et al., 1999	6	lexical decision	English	fMRI	block	word (N)	visual	abstract > concrete	1
[93] Kumar, 2016	20	orthographic judgment	Hindi	fMRI	block	word (N)	visual	abstract > concrete	4
[94] Noppeney et al., 2004	15	semantic judgment (synonym judgment)	English	fMRI	block	word (N)	visual	abstract > concrete	4
[95] Perani et al., 1999	14	lexical decision	Italian	PET	block	word (N, ADJ, V)	visual	abstract > concrete	7
[96] Pexman et al., 2007	20	semantic judgment (categorization)	English	fMRI	event-related	word (N, V)	visual	abstract > concrete	21
[87] Sabsevitz et al., 2005	28	semantic judgment (semantic similarity)	English	fMRI	event-related	word (N)	visual	abstract > concrete	10
[88] Straube et al., 2013	20	semantic judgment (content judgment)	German, Russian (control)	fMRI	event-related	sentence	visual or auditory	abstract > concrete	6
[23] Tettamanti et al., 2005	17	passive listening	Italian	fMRI	block	sentence	auditory	abstract > concrete	1
[89] Van Dam et al., 2016	14	passive reading	English	fMRI	event-related	sentence	visual	abstract > concrete	6
[47] Vigliocco et al., 2014	20	lexical decision	English	fMRI	block	word (N)	visual	abstract > concrete	2
[90] Wallentin et al., 2005	18	semantic judgment (comprehension)	Danish	fMRI	block	sentence	visual or auditory	abstract > concrete	23
[91] Wilson-Mendenhall et al., 2013	13	semantic judgment (concept-scene match)	English	fMRI	block	word (N, V)	visual	abstract > concrete	4

**Table 2:** Results for the conjunction analysis. Results are reported in Montreal Neurological Institute (MNI) space with anatomical labels from the Automated Anatomical Atlas (AAL). L = left; R = right.

Abstract ∩ Concrete								
Cluster N	Cluster Volume in mm^3^	AAL Region	x	y	z	Peak ALE Value	*p*-Value	Z Score
1	3176	Temporal_Mid_L	−50	−66	20	0.010	//	//
1		Temporal_Mid_L	−52	−60	10	0.008	//	//
1		Temporal_Mid_L	−54	−60	20	0.008	//	//
1		Angular_L	−52	−58	30	0.007	//	//
1		Temporal_Mid_L	−54	−68	10	0.007	//	//
2	3112	Precentral_L	−36	−4	60	0.009	//	//
2		Postcentral_L	−38	−20	50	0.009	//	//
2		Precentral_L	−38	−12	54	0.009	//	//
2		Precentral_L	−46	0	52	0.009	//	//
2		Precentral_L	−42	−4	52	0.008	//	//
2		Postcentral_L	−44	−22	54	0.008	//	//
2		Precentral_L	−36	−6	64	0.008	//	//
2		Precentral_L	−30	−2	58	0.008	//	//
3	1040	Temporal_Mid_L	−64	−38	0	0.007	//	//
3		Temporal_Mid_L	−56	−34	−2	0.007	//	//
3		Temporal_Mid_L	−52	−38	−6	0.006	//	//
3		Temporal_Mid_L	−64	−46	−2	0.006	//	//
3		Temporal_Mid_L	−58	−28	−2	0.006	//	//
4	960	Frontal_Med_Orb_R	4	52	−8	0.009	//	//
4		Frontal_Med_Orb_L	−6	50	−14	0.009	//	//
5	952	Rolandic_Oper_L	−48	−18	16	0.012	//	//
6	896	Frontal_Inf_Orb_L	−30	28	−6	0.008	//	//
6		Frontal_Inf_Tri_L	−34	26	0	0.006	//	//
7	840	Temporal_Inf_L	−48	−54	−14	0.009	//	//
7		Occipital_Inf_L	−44	−64	−12	0.005	//	//
8	816	Frontal_Inf_Tri_L	−48	18	26	0.007	//	//
8		Precentral_L	−48	12	30	0.007	//	//
8		Precentral_L	−48	6	36	0.006	//	//
9	632	Occipital_Mid_L	−24	−88	0	0.009	//	//
10	576	Frontal_Inf_Orb_L	−46	30	−16	0.009	//	//
11	384	Temporal_Mid_L	−54	−4	−22	0.008	//	//
12	384	Insula_L	−40	6	0	0.009	//	//
13	360	Supp_Motor_Area_L	−6	4	60	0.008	//	//
14	336	SupraMarginal_R	54	−28	18	0.006	//	//
14		Temporal_Sup_R	60	−36	16	0.005	//	//
15	280	Precentral_L	−50	4	42	0.008	//	//
16	280	Frontal_Mid_L	−30	22	46	0.009	//	//
17	264	SupraMarginal_R	56	−40	34	0.007	//	//
18	256	Temporal_Mid_L	−62	−22	−14	0.009	//	//
19	232	Frontal_Inf_Orb_L	−34	24	−18	0.007	//	//
20	216	Temporal_Mid_L	−60	−40	10	0.007	//	//
21	176	Cingulum_Ant_R	2	11	−13	0.006	//	//

**Table 3:** Results for the concrete > abstract comparison. Results are reported in Montreal Neurological Institute (MNI) space with anatomical labels from the Automated Anatomical Atlas (AAL). L = left; R = right.

Concrete > Abstract								
Cluster N	Cluster Volume in mm^3^	AAL Region	x	y	z	Peak ALE Value	*p*-Value	Z Score
1	2344	Parietal_Inf_L	−36	−80	40	0.018	<0.00001	4.67
1		Parietal_Inf_L	−28	−70	48	0.015	<0.0001	4.16
1		Angular_L	−38	−72	46	0.014	<0.0001	3.94
1		Occipital_Mid_L	−42	−74	34	0.011	<0.001	3.45
1		Angular_L	−50	−70	38	0.010	<0.001	3.19
2	1856	Fusiform_L	−26	−38	−18	0.020	<0.000001	5.11
2		ParaHippocampal_L	−24	−32	−22	0.018	<0.00001	4.68
3	1608	Angular_R	44	−68	34	0.021	<0.0000001	5.29
4	1112	Cingulum_Post_R	8	−54	12	0.020	<0.000001	5.07
5	1048	Temporal_Mid_L	−56	−62	2	0.022	<0.0000001	5.47
6	952	Precuneus_L	−10	−56	12	0.016	<0.00001	4.47
7	696	Frontal_Inf_Tri_L	−38	30	16	0.019	<0.000001	4.96
8	512	Frontal_Inf_Tri_L	−44	44	6	0.016	<0.00001	4.31
9	488	Frontal_Mid_L	−28	26	46	0.015	<0.0001	4.18
10	280	SupraMarginal_L	−62	−32	30	0.013	<0.001	3.72
11	264	Frontal_Inf_Oper_L	−54	12	14	0.012	<0.001	3.70
12	248	Temporal_Inf_L	−48	−56	−16	0.015	<0.001	4.14
13	216	Precuneus_R	4	−64	30	0.012	<0.001	3.66
14	200	Cingulum_Mid_L	−6	−38	38	0.013	<0.001	3.75

**Table 4:** Results for the abstract > concrete comparison. Results are reported in Montreal Neurological Institute (MNI) space with anatomical labels from the Automated Anatomical Atlas (AAL). L = left; R = right.

Abstract > Concrete								
Cluster N	Cluster Volume in mm^3^	AAL Region	x	y	z	Peak ALE Value	*p*-Value	Z Score
1	5992	Frontal_Inf_Tri_L	−52	20	4	0.030	<0.0000000001	6.55
1		Temporal_Pole_Sup_L	−52	10	−20	0.019	<0.000001	4.89
1		Frontal_Inf_Orb_L	−48	18	−10	0.016	<0.00001	4.34
2	2080	Temporal_Mid_L	−50	−30	−4	0.017	<0.00001	4.61
2		Temporal_Mid_L	−50	−36	−2	0.017	<0.00001	4.59
3	1032	Precuneus_R	2	−54	28	0.020	<0.000001	4.99
4	760	Frontal_Sup_Medial_L	−8	56	30	0.020	<0.000001	5.01
5	696	Temporal_Pole_Mid_R	52	12	−24	0.014	<0.0001	3.97
6	648	Temporal_Inf_L	−50	6	−36	0.015	<0.0001	4.19
7	264	Temporal_Sup_L	−56	−42	22	0.012	<0.001	3.57
8	168	Lingual_L	−16	−88	−10	0.012	<0.001	3.52
